# Meetings of Societies

**Published:** 1920-12

**Authors:** 


### MEETINGS OF SOCIETIES.

### Bristol Medico-Chirurgical Society.

*Annual Meeting, October 13th, 1920.*

After a vote of thanks to the retiring President, Dr. I. Walker Hall, had been enthusiastically carried, Dr. L. E. V. Every-Clayton took the chair and gave an introductory address on **Medicine and Surgery** (*see* p. 193). Later a cordial vote of thanks was given to Dr. Every-Clayton for the able and nteresting address he had delivered.

The Secretary, Mr. A. J. Wright, and the Editorial Secretary, read their Annual Reports. The Honorary Librarian, Mr. C. K. Rudge, read the Library Report, which was as follows :—

It is well from time to time to take stock of our Library and to note its growth and the extent to which its efficiency has been maintained. The ten years from 1910 to 1920 have been selected as a suitable period to pass in review. During that period 2,646 volumes have been added to the Library of the Medico-Chirurgical Society, and 884 volumes to the University Library, making the total accessions 3,530. The majority of these are new books or bound periodicals. Members will note that this decade embraces the five years of the Great War. The Library Committee may be congratulated on their success in adding so large a number of books to the Library, and at so small a cost, on an average that of £25 a year, including binding and a few other expenses. During this period 5,689 volumes have been borrowed. The services of Mr. Farr, the Clerk, are much appreciated by all those who frequent the Library.

The Library of the Medico-Chirurgical Society now contains 14,885 bound volumes. During the year 231 volumes have been added. These have been received from the following sources :—

| | | | |
|---|---|---|---|
| Sent for Review | .. .. | 74 | volumes. |
| By Exchange | .. .. | 35 | ,, |
| Presented | .. .. | 108 | ,, |
| Periodicals | .. .. | 14 | ,, |

During the year 50 volumes of the more important periodicals have been bound, but owing to the high cost of binding there remain 250 volumes unbound.

The Library is in regular receipt of 102 periodicals; 5 new journals have been added to the exchange list, and 9 resumed since the war.

For the convenience of Members the hours during which the Library remains open have been extended since May to 7 p.m.

The great need of money for the maintenance and development of the Library was emphasised.

The following are the officers of the Society for the Session 1920–1921: President—L. E. V. Every-Clayton, M.D. Lond.; President-Elect—Cyril H. Walker, M.B. Cantab. Committee (*ex officio*)—I. Walker Hall, M.D. Vict. (retiring President); P. Watson-Williams, M.D. Lond. (Editor of *Journal*); C. K. Rudge, M.R.C.S. (Hon. Librarian); J. M. Fortescue-Brickdale, M.D. Oxon. (Editorial Secretary); W. S. V. Stock, M.B. Lond. (Hon. Secretary). *Elected*—R. Shingleton-Smith, M.D. Lond.; R. G. P. Lansdown, M.D.; J. Lacy Firth, M.S. Lond.; J. O. Symes, M.D. Lond.; D. C. Rayner, F.R.C.S.; T. Carwardine, M.S. Lond.; Forbes Fraser, F.R.C.S.; J. A. Nixon, C.M.G., M.D. Library Committee—C. K. Rudge,. M.R.C.S.; W. A. Smith, M.D.; George Parker, M.D.

**INCOME AND EXPENDITURE ACCOUNT OF THE BRISTOL MEDICO-CHIRURGICAL SOCIETY, SESSION 1919–1920.**

| Receipts. | £ | s. | d. | Expenditure. | £ | s. | d. | £ | s. | d. |
|---|---|---|---|---|---|---|---|---|---|---|
| Balance from 1918–1919 | 187 | 2 | 2 | University of Bristol .. | | | | 20 | 0 | 0 |
| Members' Subscriptions | 268 | 3 | 0 | Honoraria— | | | | | | |
| Interest on Deposit .. | 2 | 19 | 9 | Library Clerk .. .. | | | | 13 | 0 | 0 |
| | | | | University Marshal .. | | | | 2 | 10 | 0 |
| | | | | To *Journal* .. .. .. | | | | 129 | 7 | 6 |
| | | | | Audit Fee .. .. .. | | | | 1 | 1 | 0 |
| | | | | Miscellaneous: | £ | s. | d. | | | |
| | | | | Fire Insurance | 1 | 4 | 0 | | | |
| | | | | Postage .. | 3 | 0 | 0 | | | |
| | | | | Expenses Clinical Meeting .. | 1 | 15 | 0 | | | |
| | | | | Printing and Stationery .. | 12 | 14 | 9 | | | |
| | | | | Lantern .. | 0 | 10 | 6 | | | |
| | | | | | | | | 19 | 4 | 3 |
| | | | | Balance in Bank | 166 | 15 | 8 | | | |
| | | | | Deposit A/c .. | 106 | 6 | 6 | | | |
| | | | | | | | | 273 | 2 | 2 |
| | £458 | 4 | 11 | | | | | £458 | 4 | 11 |

*November 10th*, 1920.

L. E. V. Every-Clayton, M.D., President, in the Chair.

Members of the University of Bristol Speleological Society gave an account of the work undertaken during the past year, including :—

1. An account of the Palæolithic Remains from "The Cave," Burrington, by Prof. E. Fawcett.

2. An account of Early British Remains from "The Keltic Cavern," by Mr. L. S. Palmer, M.Sc.

Professor Fawcett exhibited and described the more important objects found in the cave commonly known as Aveline's Hole, and gave a short account of the history of the cave since it became known as a bone-cave. The cave is situated near Burrington in the Mendips.

The objects found included parts of the human skeleton, flint weapons and implements, a harpoon, and about three dozen perforated shells, including an oyster shell likewise perforated. In addition to these there were bones of many animals, including the left half of the mandible and part of the maxilla, together with remains of the antlers of an enormous extinct red-deer; there were two mandibles of the brown bear, part of the skull of a large cat (? wild cat), and several mandibles of the arctic lemming.

Of the human remains one calvaria was practically complete, and had articulated with it the right malar bone. This skull was of the long-headed, *i.e.* dolichocephalic type, its breadth index being 70.3. The height index was practically 74. The general characters of the skull point to its being of the female sex, and from the fact that the sagittal suture was synostosed the age may be taken as somewhat over 50, if skulls of this period behave as modern ones do. This skull was found partly embedded in and partly below a thick stratum of stalagmite, perhaps eight inches in thickness.

One of the most striking features exhibited by the cranium was the marked festooning of the supratemporal crest, which, as it crossed the coronal suture shot up suddenly at an angle of 30 degrees before taking a backward course. Another calvaria, which, though incomplete anteriorly, appeared to be brachycephalic, was of the greatest interest because it indicated the presence of two races inhabiting the cave at the same time, both skulls having been found under and in the same stratum of stalagmite, but on opposite sides of the cave.

The implements found were ordinary flakes together with many micro-liths. The association of microliths with dolicho-ecphalic and brachycephalic skulls is of great interest, occurring, so far as is known, only elsewhere at Ofnet, near Munich, and is characteristic of the Azilian period, *i.e.* at the very end of the Palæolithic period or even the transition between Palæolithic and Neolithic. The harpoon was of great beauty, made of red-deer antler and closely allied to the Magdalenian horn, but flat in the shaft, not cylindrical. No incised bones and paintings were seen, nor were such to be expected at this period. Amongst the limb-bones of man a tibia showed marked platycnemia, *i.e.* it was sabre-like in horizontal section, and the facet on the head for the external condyle of the femur was markedly convex from before backwards.

Large numbers of fragments of human bones were found, but so broken up that it was impossible to piece them together.

Of the mammalian bones the most strikingly interesting was the half-mandible of the red-deer, whose teeth showed a well-marked cuspidate cingulum, and as evidence of the climate of the time the presence of mandibles of the arctic lemming speak with some certainty.

Mr. L. S. PALMER, M.Sc., gave an account of **"The Keltic Cavern"** which was discovered in September, 1919, by the University of Bristol Speleological Society. Since that date the cave has been surveyed and the floor excavated. This work, which has been in progress for a year, has led to the interesting conclusion that at some time between 400 B.C. and the present era the cave was inhabited for a short time by a tribe of early British settlers, known as the Brythons, who invaded this country from the shores of northern France. These same people built Glastonbury Lake Village, and are known to have inhabited Wookey Hole, Worlebury Camp, and some hut circles on Brean Down, all of which places are within a few miles of the cave in question.

A unique feature of this Mendip cave—which has been aptly named "The Keltic Cavern"—is the fact that no evidence of earlier occupation has resulted from an investigation of the deeper strata of the floor, whilst there is no trace of Roman occupation in the upper layers. The evidence for the Brythonic occupation was found in the form of pottery, iron implements, bronze ornaments, worked bone and stone and many bones of domestic and wild animals, all typical of the late Keltic period.

All these finds were deposited either on the surface of the cave floor or in a thin black band of mud which constituted the uppermost layer. In most cases a thin coating of stalagmite covered the superficial material.

The most interesting finds are bronze hubs, bands of chariot wheels, bronze bracelets and finger rings, iron slave shackles, an iron key, portion of a currency bar, etc., whilst cheek-pieces, spindle-whorls and a stone grinder are amongst other examples of worked material. The design and technique of the pottery is comparable with that found in most late Keltic settlements.

Only three human bones were discovered, one of which possibly shows anatomical features comparable with the human bones from the Brean Down hut circles.

There is considerable evidence to show that the cave was occupied under abnormal circumstances, and from other associated facts it has been concluded that the cavern was used by the Brythons as a temporary refuge, possibly at some period during the first half of the Early Iron Age.

Votes of thanks were passed to Professor Fawcett and Mr. Palmer, with cordial expressions of appreciation of the valuable work being carried on by the Speleological Society. The Society was congratulated on the possession of a "research laboratory" close at hand of such dimensions, so rich in remains as the limestone hills encircling Bristol.

---

*December 8th*, 1920.

L. E. V. Every-Clayton, M.D., in the Chair.

Dr. Cecil Williams showed (1) a case of **Periodic Jaundice in a child aged 5½ years.** The attacks occur about once a year, and last several months. The present attack began two months ago with deep jaundice. The stools were putty-coloured and contained an excess of bile, the skin was very irritable. The liver is definitely enlarged but the spleen is not. The red corpuscles are 4,500,000 per cmm., the leucocytes 15,000, the small lymphocytes constituting 57 per cent. The Wassermann reaction was negative. The child is beginning to improve.

(2) A case of **Purpura Hæmorrhagica.** The boy, aged 12¾ years, had never been strong since diphtheria at five. The family history was negative. All over the body there were purpuric spots of various sizes. He had well-marked pyorrhœa alveolaris, but a swab from the gums was sterile. Neither spleen nor liver could be felt. The blood film was not abnormal. The coagulation time was 7½ minutes. After a rectal injection of 10 c.c. of anti-streptococcus serum there was a fresh outcrop of spots and sharp abdominal pain. The boy then developed peculiar mental delusions. This was succeeded in a fortnight's time by another crop of spots. 40 c.c. of the mother's serum were then given subcutaneously. Since then the boy had improved and no further spots had been seen. Three weeks after 4 c.c. of the father's serum was injected, and the improvement has been maintained.

Dr. Carey Coombs then read a paper on **Persistence of the Ductus Arteriosus,** with notes of four cases, in one of which the diagnosis was confirmed by autopsy. In this case death was due to an intercurrent endocarditis spreading from the pulmonary artery to the aortic and pulmonary and mitral valve. This had manifested itself in life by a hectic fever, wasting, splenomegaly, petechiæ, and hæmaturia. The ductus was found to be a short wide channel opening from the pulmonary artery at its bifurcation into the origin of the descending aortæ. In all the cases the most striking physical sign was the characteristic bruit heard over the left upper chest and left upper back. It began with the onset of ventricular systole and ran on to the middle of diastole. This was illustrated by a phonocardiographic record from one of the cases. Another fact observed in three of the cases was that this murmur was accompanied by a thrill, the systolic part of which could be felt in the arteries of the neck. Skiagraphy showed the shadow which had been described by Gerhardt extending up from the normal cardiac shadow along the left border of the manubrium, but this was to be regarded as of less diagnostic value than the murmur. Electro-cardiographic and other evidence was brought to show that even in the presumed absence of any pulmonary stenosis this anomaly might be accompanied by a small degree of right ventricular hypertrophy. The absence of cyanosis and of definite polycythæmia, and the relatively favourable character of the prognosis were alluded to. The average age of the four patients when last heard of was 27.

Mr. Rendle Short read a paper on **The Causation of Appendicitis.** He began by quoting figures from the Bristol Royal Infirmary to show that in recent decades there had been a sharp rise in the incidence of the disease. The reality of this rise, which was most apparent in the years 1895–1905, was then examined, and it was shown by reference to the records of the years preceding the rise that there had been no overlooking of the disease. It was also argued from the available facts that in these earlier years the absence of cases of appendicitis could not be ascribed to failure on the part of the family practitioner to send the cases to hospital. The rise in incidence was therefore a real one. The distribution of the disease in the human community was next examined and a number of interesting data adduced in favour of the view that its incidence is lower in communities that live on a relatively simple diet. This view was supported by a further comparison of its occurrence in wild animals in captivity, and the apparent freedom of similar animals when living in a wild state. Having argued that the increase in the disease was due to dietetic changes, Mr. Short proceeded to analyse a large amount of statistical evidence by way of discovering the particular dietetic change that should be held responsible. The fact which showed the closest parallelism to the curve of appendicitis incidence was the increase in the importation of meat. This fact was examined at length and explained not as proving a direct relation between the amount of meat consumed and the liability to appendicitis, but as suggesting that a diet comparatively rich in meat and therefore comparatively poor in cellulose, did probably pre-dispose to the disease.

A number of members took part in the discussion that followed each of these papers.

### South-Western Ophthalmological Society.

The inaugural meeting of this Society was held at the Bristol Eye Hospital on October 22nd, 1920.

The Society consists of more than fifty members. It is hoped that the scope of its proceedings will be of sufficiently wide interest to attract not only oculists but others, especially general practitioners and physicians. The cases shown and the discussions will, so far as is possible, be arranged with this aim in view.

At the inaugural meeting the following officers were elected: President—Mr. F. Richardson Cross (Bristol). Vice-Presidents—Mr. A. C. Roper (Exeter), Mr. R. J. Coulter (Newport). Secretary—Mr. E. H. E. Stack (Bristol). Committee—Mr. A. W. Prichard (Bristol), Mr. H. H. Du Boulay (Weymouth), Mr. J. Burdon-Cooper (Bath), Mr. C. E. S. Flemming (Bradford-on-Avon), Mr. D. Leighton-Davies (Cardiff), Mr. R. Jaques (Plymouth).

At the morning session cases were shown. The members then lunched together and in the afternoon Mr. Roper read a paper on "The Causes of Iritis."

